# Flavor Profile Evaluation of Soaked Greengage Wine with Different Base Liquor Treatments Using Principal Component Analysis and Heatmap Analysis

**DOI:** 10.3390/foods12102016

**Published:** 2023-05-16

**Authors:** Peipei Zhao, Chang Liu, Shuang Qiu, Kai Chen, Yingxiang Wang, Caiyun Hou, Rui Huang, Jingming Li

**Affiliations:** 1 CAU Sichuan Chengdu Advanced Agricultural Industrial Institute, Chengdu 611430, China; z15355952943@163.com; 2 College of Food Science & Nutritional Engineering, China Agricultural University, No. 17 Tsinghua Dong Road, Beijing 100083, China; 3 College of Food Science & Technology, Henan Agricultural University, No. 63 Nongye Road, Zhengzhou 450002, China; 4 Sichuan Mehe Wine Industry Co., Ltd., No. 551 Xiling Avenue, Jinyuan Town, Dayi County, Chengdu 611330, China; 5 Luzhou Laojiao Co., Ltd., Luzhou 646000, China

**Keywords:** greengage wine, gas chromatography–mass spectrometry, aroma-active compounds, sensory evaluation, correlational analysis

## Abstract

The selection of base liquor plays a crucial role in the flavor of soaked greengage wine. This study aimed to investigate the effects of different base liquor treatments on the physicochemical characteristics and aroma composition of greengage wine. We carried out a comprehensive analysis using HPLC for the determination of organic acids and GC-MS for the determination of volatile aroma compounds, combined with sensory evaluation. The results showed that the red and yellow colors were the darkest in the high-alcohol group, while the citric acid content was the highest in the sake group (21.95 ± 2.19 g/L). In addition, the greengage wine steeped in 50% edible alcohol had more terpenes, a significantly higher concentration of acid–lipid compounds, and a more intense aroma compared to that of the low-alcohol group, whose typical aroma compounds were greatly reduced. The sensory results showed that the greengage wine treated with baijiu had a distinct alcoholic flavor, while almond flavors were more intense in the greengage wine treated with 15% edible alcohol. In this study, base liquor was used as the main influencing factor to provide new research ideas for the flavor optimization of soaked greengage wine.

## 1. Introduction

Greengage (*Prunus mume*) is a plant belonging to the Rosaceae family that is rich in bioactive substances and possesses several healthcare benefits [1]. According to the literature, fresh greengage fruit contains large amounts of protein, fat, many essential amino acids, organic acids, and minerals [2]. In addition to these essential nutrients, greengage contains phenolic substances such as squalene, epicatechin, chlorogenic acid, and rutin, which have antibacterial effects [3,4]. Processed greengage fruit can also be used for the treatment of cough, vomiting, diarrhea, and fever [5]. Mature greengage is mainly used for the production of alcoholic beverages and dried fruit [4]. Currently, greengage wine is popular in Asia due to its unique flavor and nutritional properties, including a low alcohol content [6].

The greengage wine on the market at present can be mainly divided into two types: fermentation and soaking [7]. The former is brewed by adding yeast, while the latter is brewed by soaking the greengage in a base liquor. Related studies show that soaking greengage wine improves fruit flavor more than fermentation [6,8]. Compared with soaked greengage wine, the flavor of fermented greengage wine tends to be more diverse due to the complex fermentation process and byproducts of microbial metabolism, which cause it to lose its unique characteristics and style [9,10]. In most industrial production practices, soaking is a very common method as it is the most economical and easy way to maintain the flavor of fruit wine [11]. After the soaking process, the wine will form a unique flavor and body [6]. South Korea and Japan mainly use 15–18%*v/v* sake as the base liquor for soaking. In China, however, Baijiu (38–65%*v/v*) or edible alcohol (75–95%*v/v*), which is brewed locally, is more commonly used [12].

According to the International Organization of Vine and Wine, wine is generally defined as the product obtained from the fermentation of grape juice by yeast followed by aging; however, the term also applies to fermented beverages made from other fruits or vegetables, generally with an alcohol content of 5–13%*v/v* [13]. Flavor is an important attribute of fruit wine, including aroma, taste, and sense, and is also the main factor determining consumer preference and acceptance [14,15]. Many factors affect the quality of greengage wine. In addition to the main external factors, such as geographical environment, climate, and cultivation technology [16], the conditions selected for processing will also have a considerable impact on the content and quality of flavor compounds in the wine [17]. Gas chromatography–mass spectrometry (GC-MS) and meteorological chromatography-olfaction (GC-O) are commonly used for the analysis of flavor compounds, as both are efficient and highly sensitive techniques [18]. In addition, the contribution of flavor compounds to the olfactory characteristics of wine can be evaluated using odor activity value (OAV) measurements [19].

In recent years, research on the flavor of greengage wine has increased. Tiantian Tian et al. optimized the fermentation conditions of three different greengage wines using the response surface method and central composite experimental design. The results showed that there were significant differences in the flavor compounds and concentrations between the three fermented greengage wines. A total of 53, 30, and 32 flavor substances were identified from the optimized samples [20]. In several studies, researchers have found that when using local acid-resistant non-saccharomyces cerevisiae to ferment greengage, the fermented greengage wine has more esters and higher levels of polyphenols, which can improve the flavor quality and nutritional value of the wine [11,21]. In addition, the content of polyphenols in greengage at different maturity stages also varies greatly. Chang Liu et al. analyzed polyphenols at different maturity stages using ultra-high-performance liquid chromatography-electrospray ionization triple quadrupole mass spectrometry (UPLC-QTRAP-MS/MS) technology and widely targeted metabolomics technology. The results showed that polyphenols first increased and then decreased under the influence of temperature, sunshine duration, humidity, and radiation during the greengage ripening process [22].

Thus far, most research has focused on fermented greengage wine, and studies on soaked greengage wine are less common. The existing research shows that by using ultrasonic-assisted treatment, the aging speed of soaked greengage wine can be greatly accelerated and the content of fusel oil and alcohol compounds in the wine can be significantly reduced, while the content of acid ester compounds can be significantly increased [6]. Tiantian Tian et al. used partial least squares regression analysis to model and analyze the relationship between the flavor-active compounds, aromatic compounds, and sensory properties of 20 greengage wines sold on the market, revealing the relationship between chemical components and sensory properties [20]. However, there have been no reports on the effect of the base liquor on the flavor quality of soaked greengage wine. Therefore, in this study, odor activity value (OAV) combined with PLS-DA was used to evaluate the effect of the key volatile compounds of the base liquor on the aroma of greengage wine. In addition, the interaction between volatile compounds and the sensory evaluation of greengage wine was also determined. The results of this study will provide a useful guide for improving the functional characteristics and quality of soaked greengage wine.

## 2. Material and Methods

### 2.1. Preparation of Greengage

Nangao greengage (*Prunus mume*) was collected from the Xiling Greengage Valley (30°25′ N, 102°50′ E), Chujiang Town, Dayi County, Chengdu, Sichuan Province, China in June 2021. Fruits with intact skins were selected for the study, and whole fruits were transported to the laboratory 48 h after fresh picking. Subsequently, the samples were immediately cleaned, pedicled, soaked, and bottled. Chrysanthemum authentic sake (15%*v/v*), manufactured by the chrysanthemum authentic wine factory club, was purchased from Jingdong’s self-operated flagship store. Edible alcohol (75%*v/v*) was purchased from the Jingdong Naihui Medical Device Store.

### 2.2. Soaked Greengage Wine Brew

The prepared greengage fruit was poured into the fermentation tank, followed by the addition of green greengage, rock sugar, and base liquor in a ratio of 1:1:1. According to the experimental requirements, 15%*v/v* of edible alcohol (FS), 50%*v/v* of edible alcohol (TS), sake (SK), and baijiu (BJ) were selected as base liquors, and samples were collected every 15 days and shaken thoroughly. The changes that occurred in the greengage wine during soaking are shown in Figure S1.

### 2.3. Physicochemical Analyses

The total sugar content (g/L) was determined using the Fehling reagent [13] and the direct titration method. The pH value was measured with a pH meter in the laboratory. According to the Compilation of Chinese Food Industry Standards (2000), the total acid content in greengage wine was neutralized and titrated with 0.1 mol/L of sodium hydroxide and expressed as citric acid content (g/L) as determined by acid–base titration. The alcohol content was determined via distillation using a hydrometer.

### 2.4. CIELAB Analysis

A description of the CIELAB analysis method can be found in the existing literature [23]. The wine sample was filtered through a 0.45 µm membrane (Syringe Filter, PTFE, Superco, Bellefonte, PA, USA) and transferred to a 1 mm quartz cuvette; then, its absorbances were measured at 440, 530, and 600 nm. Subsequently, the corresponding color characteristic parameters were calculated. Distilled water was used as the blank control during determination.

### 2.5. Organic Acids in Greengage Wine

Organic acids were analyzed using a Shimadzu Prominence LC-20A HPLC system (Shimadzu, Kyoto, Japan) equipped with a DAD detector and a TechMate C18-ST analytical column (4.6 × 250 mm, 5 μm) maintained at 21 °C, where the mobile phase A was 0.02 M diammonium dihydrogen phosphate solution. The pH of the sample was adjusted to 2.5 with phosphoric acid. The flow rate, detection wavelength, and injection volume were 1.0 mL/min, 210 nm, and 10 μL, respectively. The samples were filtered through a 0.22 μm filter prior to injection. The calibration curves of seven organic acid standards were plotted for quantitative analysis (Table S1).

### 2.6. Headspace Solid-Phase Microextraction (HS-SPME)

A DVB/CAR/PDMS extraction head (Supelco, Bellefonte, PA, USA) was aged at the gas-phase sampling port (270 °C) for 30 min before use. Aliquots of 5 mL of clarified greengage wine sample, 10 μL of 4-methyl-2 amyl alcohol (1024 mg/L) dissolved in ethanol, and 2.02 g of NaCl were placed in a 10 mL headspace sample injection bottle before the rotor was added. The polyethylene bottle cap with a polytetrafluoroethylene (PTEE) silicon spacer was tightened and the sample bottle was placed on a magnetic stirring table. The sample bottle was maintained at 45 °C for 30 min until the gas and liquid phases reached equilibrium. A pre-treated SPME fiber was inserted into the headspace bottle, placed 1 cm above the liquid level, and extracted for 30 min at a speed of 400 r/min. When the gas, liquid, and solid phases in the headspace bottle reached equilibrium, the extraction fiber was removed from the sample bottle and inserted into the gas chromatography–mass spectrometry (GC-MS) sample inlet to conduct thermal analysis for 8 min in non-split flow mode. Three replicates were conducted for each sample.

### 2.7. Determination of Volatile Organic Compounds (VOCs) Using GC-MS

An Agilent 7890B gas chromatograph and an Agilent 5977B mass spectrometer were used to analyze the aroma substances. The gas chromatograph was loaded with an ultra-fine capillary column (HP-INNOWAX with dimensions of 60 m × 0.25 mm × 0.25 μm, Agilent, J&W Scientific, Folsom, CA, USA). High-purity helium (He > 99.999%) was used as the carrier gas with a flow rate of 1 mL/min, and SPME was used for manual injection without split flow. The injection port temperature was 250 °C and the thermal analysis time was 8 min. The temperature increase procedure for the column temperature box was as follows: the temperature was maintained at 40 °C for 5 min, then increased to 200 °C at a rate of 3 °C/min, and finally maintained for 2 min. The mass spectrometry ionization mode was electron ionization; the ion source temperature, ionization energy, fourth-stage rod temperature, mass spectrometry interface temperature, and mass scanning range were 230 °C, 70 eV, 150 °C, 280 °C, and 30–350 u, respectively.

The analysis was conducted using the offline software Agilent Chemical Workstation (Agilent Technologies, Inc., J&W Scientific, Folsom, CA, USA). For the qualitative analysis, the detected substances were compared to the retention index in the NIST17 database and the mass spectrum ion fragment information. The retention index of each substance was calculated using normal alkanes (C8-C40), and the values obtained were compared with those in the literature.

### 2.8. Odor Activity Value

In fruit wine brewing, not all aroma components affect the aroma and sensory properties of the wine. To further identify differences in the aroma substances of greengage wines treated with different base liquors, in combination with the threshold values of aroma compounds reported in the literature, the concept of OAV was used to determine the components contributing to the aroma. The calculation formula for OAV is OAV = c/t, where “c” is the total concentration of each flavor compound in the sample and “t” is the odor threshold of the compound in the 11%*v/v* water/ethanol solution. If OAV ≥ 1.0, the substance has an actual contribution to the aroma; otherwise, it has no contribution. The OAV of each substance was calculated according to the qualitative and quantitative results of the GC-MS analysis.

### 2.9. Sensory Analysis

Forty sensory personnel from the China Agricultural University were trained and assessed, and 25 sensory evaluators were selected to form a sensory evaluation team (15 women and 10 men, aged 20–25 years) to conduct a quantitative descriptive sensory analysis of the greengage wine samples. The greengage wine samples were scored on a 10-point scale (0–9) on the three aspects of appearance, aroma, and taste. The ranges were defined as follows: 0–2 indicates very weak variety, 3–5 indicates medium variety, and 6–9 indicates very strong variety. The evaluation was conducted in a well-ventilated, odorless, and noiseless sensory evaluation room, and mouthwash was provided to the sensory evaluation team during the evaluation to avoid the influence of aftertaste.

### 2.10. Statistical Analysis

Excel 2019 (Microsoft, Washington, DC, USA) was used for data calculation and Duncan’s test and t-test in IBM SPSS (version 25.0; SPSS Inc., Chicago, IL, USA) were used for one-way ANOVA for the analysis of significant differences, *p* < 0.05. The R language and MetaboAnalyst 5.0 (http://www.metaboanalyst.ca (accessed on 22 September 2022.)) software were utilized for principal component analysis (PCA) and mapping, and the remaining figures were drawn using Origin Pro 2018.

## 3. Results and Discussion

### 3.1. Basic Physicochemical Characteristics

Table 1 presents the relevant physical and chemical parameters for the four treatment methods. After soaking, significant differences were observed in the total sugar, total acid, alcohol, and pH among the four treatment groups. This is noteworthy because the balance of sweet and sour is essential for fermented drinks. In the low-alcohol treatment group, compared to SK, edible alcohol had a higher total acid content, which has an important impact on the flavor of the fruit wine [24] and may be related to the brown sediment formed after the polymerization of phenolic compounds [25]. The TA also reflects to some extent the quality of fruit wine and is one of the most important indicators [26]. No significant differences were observed in the total sugar, alcohol content, and pH. In the high-alcohol treatment group, significant differences were observed in the total sugar, total acid, and alcohol content between edible alcohol and BJ; however, no significant difference was observed in the pH. Overall, the total sugar content of the high-alcohol treatment group was lower than that of the low-alcohol treatment group, whereas the total acid content was higher. FS had the lowest pH, followed by SK and BJ; however, a significant difference in pH was observed between TS and BJ. As presented in Table 1, the total acid content of the four treatment groups was about 13.30–15.50 g/L, which is consistent with the relevant literature [6].

### 3.2. Analysis of Color Change during Soaking

The CIELAB color coordinate system is an objective color perception tool [24]. The results of this analysis are presented in Table 1. A statistical difference was observed between the CIELAB parameters of soaked greengage wine and wine. As presented in Table 1, FS-soaked greengage wine had the highest CIEL value. This indicated that FS-soaked greengage wine had the highest brightness but also the lowest CIEa and CIEb values, suggesting that FS has the lightest red and yellow colors. Additionally, the alcohol and liquor treatments in the high-alcohol group resulted in the highest CIEa and CIEb values, indicating that their red and yellow colors were the deepest; no significant difference was observed between them. With many foods and beverages, color is an important parameter for consumers [18] as it is usually the first sensory impression perceived. The color of wine provides information about style, maturity, production method, grape variety, growing conditions, etc. [27]. However, the influence of the base liquor on the color of soaked greengage wine has not been extensively studied and the mechanism of its action is not fully understood.

### 3.3. Organic Acids in Greengage Wine

**Figure 1:**
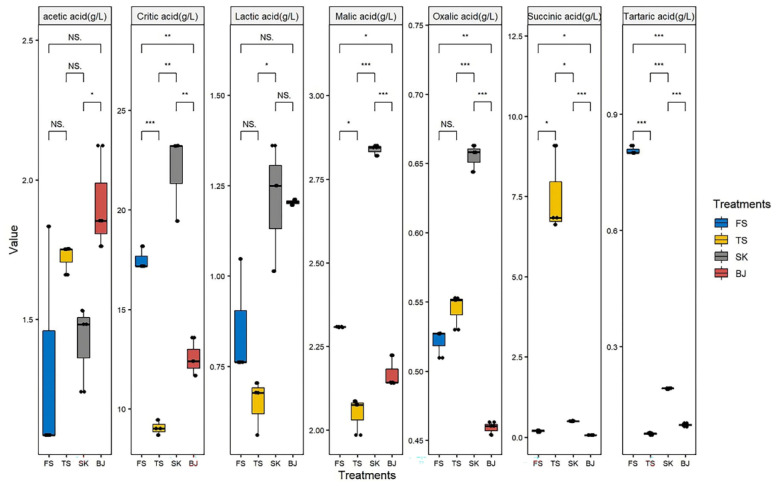
Concentration of organic acids with different base liquor soaking treatments; significance correlations are marked as *p* ≤ 0.05 (*), *p* ≤ 0.01 (**) and *p* ≤ 0.001 (***), respectively.

The levels of organic acids in the different treatment groups after soaking are shown in Figure 1. The organic acids in fruit wine have an important influence on the flavor [28], chemical stability, pH value, and thus the quality of the wine. The coordination of sour tastes depends on the composition of different organic acids and the perceived concentration level [29]. The organic acids in soaked greengage wine mainly originate from the base liquor extracted from the greengage fruit under the action of high osmotic pressure. Seven types of organic acids were detected in the soaked greengage wine. The main organic acid was citric acid, with levels up to 21.95 g/L, representing approximately 81.29% of the total organic acid content (Table 1). Citric acid was the main reason for the refreshing taste of the soaked greengage wine [30]. Malic acid is the second most abundant acid in green wines. It has a strong sour, spicy [31], and bitter taste. It is an important indicator of fruit freshness. Malic acid can increase the freshness of fruit wine by reducing the release of aroma substances. Based on previous reports, it is known that the addition of citric and malic acid enhances the intensity of flavor perception for fruit-flavored beverages and improves participants’ flavor recognition ability [32]. In this study, SK contained the highest content of citric, malic, lactic, and oxalic acids, indicating that the SK treatment was the most conducive to promoting the transformation of malic acid to organic acid during the soaking process, followed by FS, BJ, and TS. In addition, BJ had a much higher content of acetic acid than the other three groups. Acetic acid usually reacts with alcohol to form esters, conferring a negative effect on fruit wines [20]. In the present study, SK had significantly higher levels of succinic acid than the other three groups, which is also rare in previous reports.

### 3.4. Flavor Compounds

**Figure 2:**
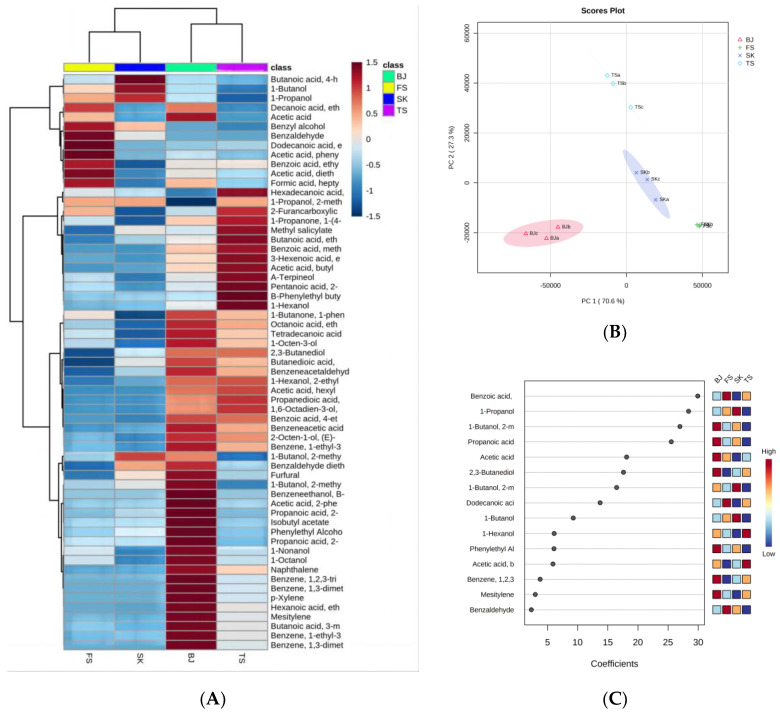
(**A**) Heatmap of flavor compounds in each treatment group during soaking. The Euclidean methodology of distance measure and the Ward clustering algorithm were selected. (**B**) Score plot of PCA for greengage wine from different treatment groups after soaking. (**C**) Ranking of characteristic esters, terpenes, and benzenes, calculated by weighting the sum of absolute regression coefficients in PLS-DA. The colored boxes on the right denote the correlations between the characteristic esters, terpenes, and benzenes and the different treatments at the end of soaking.

As presented in Table S2, 53 flavor compounds were identified in the four types of greengage wine. In addition, the aroma compounds in the four types of greengage wine were analyzed, and 51, 50, 49, and 50 aroma compounds were detected in FS, SK, TS, and BJ, respectively. There were twenty-eight esters, thirteen alcohols, seven aldehydes and ketones, three terpenes, and two other aromatic compounds [33]. To further analyze the significant differences among the four types of soaked greengage wine, we used thermographic cluster analysis (Figure 2). From Figure 2, it can be seen that the BJ treatment was conducive to the production of some alcohols and esters, such as 2-methyl-1-propanol, phenylethyl acetate, 2-methyl-1-butanol, ethyl lactate, isobutyl acetate, phenyl ethanol, 1-nonanol, 1-octanol, ethyl hexanoate, and ethyl isovalerate, which are VOCs that are highly related to the BJ treatment. In contrast, the esters produced by the TS treatment greatly improved the aroma of the fruit wine. The base liquors with a high alcohol content promoted the production of esters, which may be related to the release of aroma compounds in fruit wine due to the concentration of ethanol [34], while the VOCs responsible for the distinctive aroma and flavor of the fruit are mainly accumulated during the ripening period [35]. VOCs are biosynthesized from amino acid derivatives, fatty acid derivatives, and sugar derivatives [36]. Ethyl caproate, ethyl lactate, isobutyl acetate, phenylethyl acetate, and ethyl isovalerate, which represent sweet and fruity aromas, were high in the BJ treatment group and were also considered characteristic compounds of this group. Compared to those in the high-alcohol group, the contents of typical aroma compounds were greatly reduced in the low-alcohol group.

The relationships and differences between the treatment groups during the soaking process were studied using PCA (Figure 2B). PC1 and PC2 accounted for 70.6% and 27.3% of the variation, respectively, indicating a clear separation between the four processed samples of greengage wine. The score chart shows that components 1 and 2 accounted for 97.9% of the total variation. To better analyze the differences in the typical aroma of greengage wine treated with four different base liquors, we used PLS-DA to qualitatively characterize the greengage wine, and the coefficient of each characteristic was used to represent the overall importance (Figure 2C). Fifteen key VOCs were screened, most of which were alcohols and esters, such as 2-methyl-1-butanol, ethyl lactate, ethyl acetate, and 2,3-butanediol. These esters were highly correlated with the BJ treatment group, which was consistent with the results of the thermal polymerization diagram. Esters are often considered to be the most important component of fruit [37] and floral aromas. One source of esters is the esterification of aldehydes, alcohols, ketones, and fatty acids, while another is the metabolic synthesis of higher alcohols by microorganisms in the presence of acetyltransferases [35]. Alcohols are mainly formed during the fermentation of the original wine, while the degradation of amino acids, carbohydrates, and esters may produce fusel oils as well as floral and herbal aromas [38]. The BJ treatment group was rich in fruit alcohol and fruit ester, which is consistent with previous reports [39]. Benzaldehyde has been reported to be a characteristic aroma compound in soaked greengage wine [40]. However, in this study, its content was high only in the FS treatment group, and no significant differences were observed between the other three groups.

Not all volatile compounds affect the overall aroma of greengage wine. We used the OAV to characterize the contribution of certain aroma components to the overall aroma characteristics. When the aroma value of a certain component is greater than or equal to 1, this aroma component contributes to the aroma of the fruit wine. The higher the aroma value of a component, the greater its contribution [41]. Therefore, according to the results of PLS-DA and the components with high OAVs (Table 2), we screened 14 VOCs, including six esters, six alcohols, and two aldehydes. These were not only the key VOCs that distinguished the aroma processed by yeast but were also important contributors to the aroma characteristics of soaked greengage wine. Ethyl benzoate, isobutanol, and 2-methyl-1-butanol were the top three highly correlated VOCs; therefore, these VOCs made an outstanding contribution to the aroma of the wine, being mainly responsible for the “fruity”, “fatty”, and “banana” flavors, with the highest concentrations in the FS treatment. Although the olfactory threshold of ethyl butyrate is only 0.90 μg/L, its OAV was high, giving the greengage wine a strong apple flavor and sweet smell. Furthermore, although the threshold value of benzaldehyde is high (750.89 μg/L), it was the second richest aroma compound and had an obvious almond aroma, which is also the typical aroma of soaked greengage wine. Notably, as presented in Table 2, the almond flavor of the SK treatment group was the strongest, followed by that of the FS treatment group. Sake has been reported to contain high levels of benzaldehyde, which can increase during storage. Benzaldehyde is a benzene derivative that is mainly formed from terpenes, polyketones, and shikimate in fruits [17]. Benzaldehyde has a typically almond flavor and is one of the most important compounds affecting the overall organoleptic characteristics and consumer acceptability of greengage wine [20], generally found at a concentration of about 300 μg/L [42]. In our results, however, the level of benzaldehyde in the SK treatment group was seven times higher than that in the other groups.

### 3.5. Sensory Evaluation

**Figure 3:**
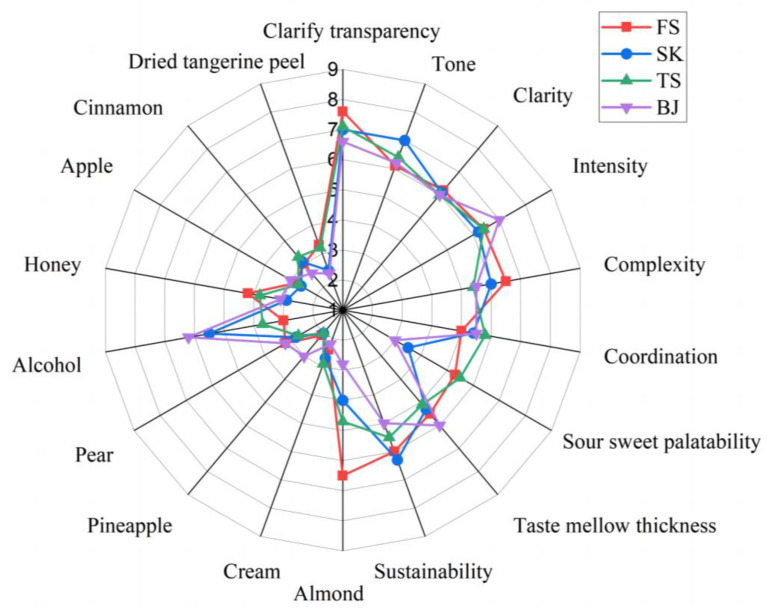
Sensory evaluation results for the four different treatment groups.

A flavor contour map drawn according to the sensory evaluation results is shown in Figure 3. The score comprised five parts: appearance, aroma, aroma sensory descriptors, overall evaluation, and taste. In terms of appearance, the clarification of greengage wine treated with FS and TS was better. In terms of aroma performance, BJ-treated greengage wine had the highest score for intensity, FS-treated greengage wine had the highest score for intensity, and TS-treated greengage wine had the highest score for coordination. In terms of clarity, there were no significant differences among the four greengage wines. In terms of taste, the greengage wine treated with SK was given a high score for persistence. The greengage wine treated with BJ had the highest score for taste alcohol thickness, that soaked with 50%*v/v* alcohol had the highest score for sour and sweet palatability, and that soaked with 15% alcohol had high scores for sour and sweet palatability, taste alcohol thickness, and persistence. In terms of taste, the score for the alcohol group was much higher than that for the commercial alcohol group, and the overall score order was TS (5.8), FS (5.7), BJ (5), and SK (4.8).

The sensory evaluation team screened the aroma of the greengage wine, and nine sensory descriptors were obtained: orange peel, cinnamon, apple, honey, alcohol, pear, pineapple, cream, and almond. Among the sensory evaluation results for the greengage wines soaked in the four base liquors, the greengage wine treated with BJ had more prominent alcohol and pineapple flavors, that treated with FS had more prominent almond and honey flavors, and that treated with TS had more prominent cinnamon and cream flavors.

### 3.6. Pearson Correlation Analysis of Aroma Sensory Attributes

**Figure 4:**
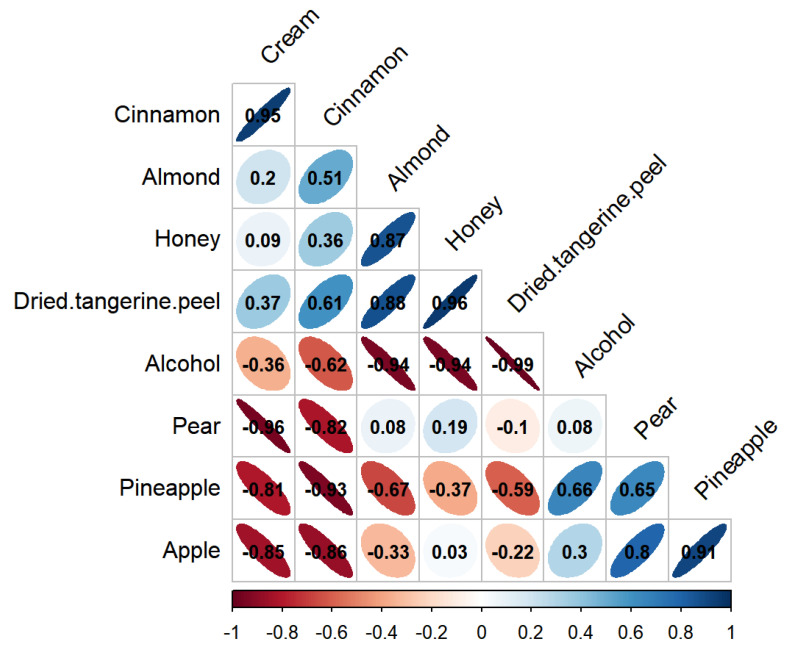
Scatter diagram showing correlation coefficients between sensory aroma attributes. The flatter the ellipse is, the larger the absolute value of the correlation coefficient is. The rounder the ellipse, the smaller the absolute value of the correlation coefficient. The direction of the ellipse’s major axis represents the positive and negative of the correlation coefficient: the upper right-lower left direction corresponds to the negative value, and the upper left-lower right direction corresponds to the positive value; The color depth indicates the correlation coefficient.

From Figure 4, it can be seen that the correlation coefficient between the butter and cinnamon flavors (r = 0.95) was large, indicating a strong correlation. The correlation coefficients between the almond, honey (r = 0.87), and orange peel (r = 0.88) flavors were large, indicating that the almond flavor had the same influence on the honey and orange peel flavors. The correlation coefficient between the honey and dried tangerine peel flavors (r = 0.87) was large, indicating a strong correlation. Notably, the three aroma sensory attributes of the soaked greengage wine, namely, the honey, almond, and orange peel flavors, are interrelated, indicating a certain degree of association. Additionally, the correlation coefficients between the apple, pineapple (r = 0.91), and pear flavors (r = 0.80) were also large.

In Figure 4, the flatter the ellipse, the greater the absolute value of the correlation coefficient. A more circular ellipse indicates that the absolute value of the correlation coefficient is small. The direction of the major axis of the ellipse represents the direction of the correlation, where the upper right-lower left direction corresponds to a negative value and the upper left-lower right direction corresponds to a positive value.

### 3.7. Correlation between Aroma-Active Compounds and Sensory Characteristics

**Figure 5:**
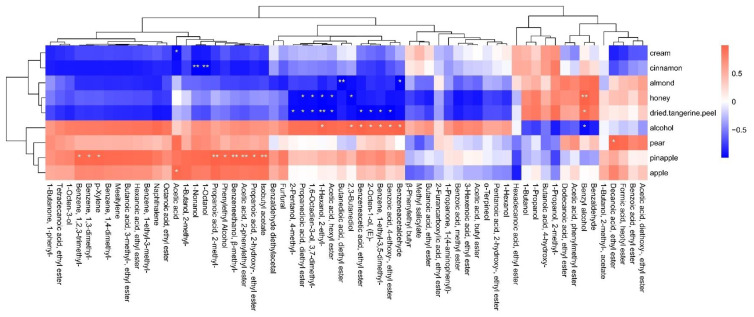
Correlation heat map between the sensory aroma attributes and GC-MS analysis results. Significant correlations are marked as *p* ≤ 0.05 (*) and *p* ≤ 0.01 (**), respectively.

Many studies have shown that some of the distinctive flavor characteristics of the wine are determined by its chemical composition; however, the exact part of the chemical composition that affects the senses of the consumer still needs to be further investigated [43]. Therefore, this study explored the association between VOCs and sensory attributes by conducting a Pearson correlation analysis between the GC-MS results and the aroma sensory evaluation results and creating a correlation heat map. From Figure 5, it can be seen that a total of 26 substances were significantly related to the aroma sensory attributes (*p* < 0.05). The aroma sensory evaluation results showed that the pineapple taste was the main aroma sensory attribute of soaked greengage wine. The substances that were significantly positively related to the pineapple taste included isobutyric acid, 2-phenyl-1-propanol, phenylethyl acetate, and isobutyl acetate, while butyl lactate was found to be negatively correlated with the pineapple taste. Acetic acid was found to have a significant positive correlation with the apple taste, ethyl decanoate had a significant positive correlation with the pear taste, and several aroma substances had a significant positive correlation with the alcohol taste, including 2,3-butanediol, ethyl phenylacetate, trans-2-octene-1-ol, ethyl p-ethoxybenzoate, and phenylacetaldehyde. Additionally, phenylethyl alcohol was positively correlated with the tastes of honey and tangerine peels.

Overall, fruit aroma was mainly positively correlated with higher alcohols and esters. Xizhen et al. [44] also found that fruit aroma had a good correlation with some esters and alcohols in strong Chinese wines.

## 4. Conclusions

In this study, the effects of different base liquors on the quality of soaked greengage wine were investigated. The influence of the base liquor on the flavor of soaked greengage wine was comprehensively evaluated via GC-MS and HPLC combined with a sensory evaluation. Our results showed that the basic physical and chemical indices of greengage wine were not significantly different after being soaked in different base liquors. The alcohol content, total acid, total sugar, and pH of the low-alcohol group were 7.44%*v/v*, 15.50 g/L, 360.09 g/L, and 3.4, respectively. Compared to the FS treatment, SK was found to significantly improve the red and yellow blending saturation of the greengage wine. In the high-alcohol group, the alcohol content, total acid, total sugar, and pH were approximately 25.74%*v/v*, 15.50 g/L, 345.21 g/L, and 3.05, respectively. There was no significant difference in chromaticity. In the low-alcohol group, malic, citric, and tartaric acids were significantly higher in the greengage wine with sake as the base liquor than in the greengage wine soaked in FS. In the high-alcohol group, the liquor significantly accelerated the leaching of citric and malic acids compared to alcohol of the same degree, and alcohol was found to soak out succinic, tartaric, and oxalic acids in greengage more than liquor. Additionally, the results of GC-MS showed that the concentrations of characteristic aroma compounds in the four kinds of soaking greengage wine treated with different base liquors were significantly different. Among them, the greengage wine treated with SK had a stronger nut flavor. In addition, the greengage wine treated with TS contained more terpenes, which can induce a floral and fruity aroma.

Additionally, a sensory evaluation experiment and Pearson coefficient correlation analysis were conducted on the greengage wines soaked in the four base liquors. The results showed that the greengage wines soaked in the four different base liquors had certain differences, among which the greengage wine soaked in TS was superior to the wine in the other three treatment groups in terms of clarity, aroma intensity, and coordination, acidity and sweetness, and overall evaluation. The fruit aroma was found to have a strong positive correlation with most esters and higher alcohols. However, the mechanism of action of the base liquor must be further verified; this is a potential direction for future research on the flavor of soaked fruit wines.

## Figures and Tables

**Table 1:** Physiochemical characteristics of soaked greengage beverage.

Treatment	FS	SK	TS	BJ
Total sugar (g/L)	360.09 ± 2.88 a	358.47 ± 6.85 a	345.21 ± 6.85 b	333.09 ± 7.88 c
Total acid (g/L)	15.50 ± 0.50 a	13.90 ± 1.10 b	13.30 ± 0.50 b	15.50 ± 0.50 a
Alcohol degree. (%*v/v*)	6.71 ± 0.13 c	7.44 ± 0.16 c	22.94 ± 0.64 b	25.74 ± 0.59 a
pH	2.70 ± 0.01 b	3.40 ± 0.73 a	2.98 ± 0.01 ab	3.05 ± 0.01 ab
CIE*L*	95.02 ± 0.57 a	93.97 ± 0.29 c	92.41 ± 1.31 b	92.14 ± 0.89 b
CIE*a*	1.58 ± 0.32 b	1.98 ± 0.07 a	2.67 ± 0.26 a	2.88 ± 0.3 a
CIE*b*	10.06 ± 0.71 c	15.14 ± 0.04 b	16.59 ± 0.16 a	17.14 ± 0.11 a
Chroma (C)	10.18 ± 0.75 c	15.27 ± 0.05 b	16.81 ± 0.12 a	17.38 ± 0.05 a
Hue angle (*h*)	81.16 ± 1.22 b	82.56 ± 0.24 a	80.85 ± 0.97 b	80.44 ± 1.02 b
Oxalic acid (g/L)	0.52 ± 0.01 b	0.54 ± 0.01 b	0.65 ± 0.01 a	0.46 ± 0.00 c
Tartaric acid (g/L)	0.81 ± 0.01 a	0.08 ± 0.00 d	0.19 ± 0.00 b	0.10 ± 0.00 c
Malic acid (g/L)	2.31 ± 0.00 b	2.05 ± 0.06 d	2.84 ± 0.02 a	2.17 ± 0.05 c
Lactic acid (g/L)	0.86 ± 0.16 b	0.65 ± 0.08 b	1.21 ± 0.18 a	1.20 ± 0.01 a
Acetic acid (g/L)	1.33 ± 0.43 b	1.72 ± 0.05 ab	1.42 ± 0.16 ab	1.91 ± 0.19 a
Critic acid (g/L)	17.5 ± 0.57 b	9.05 ± 0.39 d	21.95 ± 2.19 a	12.55 ± 0.96 c
Succinic acid(g/L)	0.19 ± 0.03 b	7.51 ± 1.37 a	0.50 ± 0.01 b	0.06 ± 0.00 b

Note: Different letters in the same row indicate significant differences (*p* ≤ 0.05).

**Table 2:** VOCs in soaked greengage beverages with OAVs higher than 1.

Category	Odor Description	Threshold (μg/L)	Treatment			
			FS	SK	TS	BJ
Phenylethyl alcohol	Floral, sweet, rosy	564.23	<1	1.52	n.d.	1.92
Benzoic acid, ethyl ester	Sweet, green, fruity, birch	55.56	7.75	9.15	5.57	6.70
Benzeneacetaldehyde	Honey, sweet, floral, chocolate	6.3	<1	1.07	n.d.	n.d.
2,3-Butanediol	Fruity, creamy	>100	2.91	1.19	n.d.	n.d.
Benzaldehyde	Sweet, oily, almond, cherry, nutty	750.89	8.67	9.48	4.72	4.91
1-Octen-3-ol	Mushroom, vegetative,	1.5	2.32	1.53	2.13	3.83
Octanoic acid, ethyl ester	Sweet, fruity, pineapple, creamy	19.3	2.20	<1	1.16	1.81
Propanoic acid, 2-hydroxy-, ethyl ester	Sweet, fruity, creamy, pineapple	50	2.40	25.94	n.d.	54.30
Hexanoic acid, ethyl ester	Sweet, pineapple, fruity, banana	5	1.54	2.34	2.34	6.47
1-Butanol, 2-methyl-	Alcoholic, fatty, cocoa	15.9	92.83	190.44	n.d.	131.66
1-Butanol	Banana	459.2	1.34	2.77	n.d.	n.d.
1-Propanol	Earthy, peanut, nutty, apple, pear	8505.6	1.24	2.15	n.d.	n.d.
Butanoic acid, ethyl ester	Fruity, sweet, apple	0.9	<1	104.73	n.d.	35.48
Isobutyl acetate	Sweet, fruity, banana	25	<1	1.42	n.d.	1.45

Note: “n.d.” means not detected.

## Data Availability

Data is contained within the article or Supplementary Material.

## Supplementary Materials

The following supporting information can be downloaded at: https://www.mdpi.com/article/10.3390/foods12102016/s1, Figure S1: Soaked process and changes in greengage fruits, including changes in fruit appearance (SK: soaked).; Table S1: Calibration curves for quantification of organic acids in the experiment. Table S2: Flavor compounds of the four soaked greengage wines.
