# Supplementary material for: Flavor Profile Evaluation of Soaked Greengage Wine with Different Base Liquor Treatments Using Principal Component Analysis and Heatmap Analysis
Source: Foods. 2023 May 16;12(10):2016. doi: 10.3390/foods12102016 (PMC10217753; doi:10.3390/foods12102016)
Supplement: Supplementary file 1 [file foods-12-02016-s001.zip › foods-2355028-supplementary.pdf]

Figure S1 Soaked process and changes in greengage fruits, including changes in fruit appearance (SK: soaked).

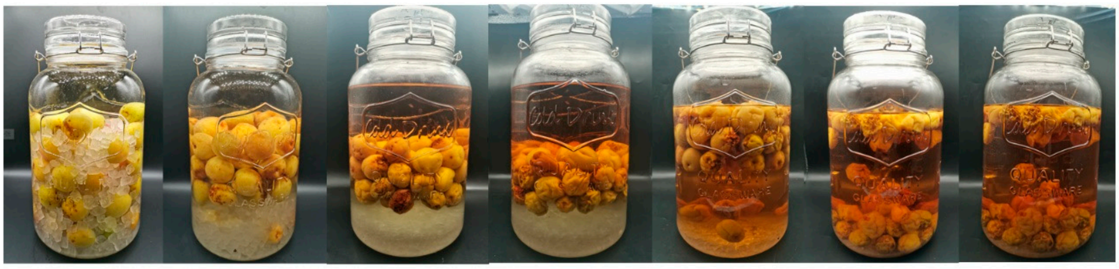

SK in 0 day | SK in 15 days | SK in 30 days | SK in 45 days | SK in 60 days | SK in 75 days | SK in 90 days

Table S1 Calibration curves for quantification of organic acids in the experiment

No.	Categories	RT (min)	Standard curves (g/L)	R^2	Range (g/L)
1	Oxalic acid	3.040	y =6,033,728.99x + 60201	0.9989	0.00525-10.68
2	Tartaric acid	3.373	y = 692570x + 8744.4	0.9979	0.00503-10.13
3	Malic acid	4.096	y = 459367x + 39085	0.9988	0.00512-50.47
4	Lactic acid	4.743	y = 295683x + 444.03	0.9999	0.00501-10.24
5	Acetic acid	5.054	y = 332676x - 405.14	0.9999	0.00527-10.65
6	Citric acid	6.610	y = 645557x + 81569	0.9998	0.005025-100.12
7	Succinic acid	7.552	y = 351044x - 870.05	1.0000	0.005015-10.20

Table S2 Flavor compounds of the four fermented greengage wines.

No.	RT (min)	Categories	RI[1]	CAS	Concentration(μg/L) FS[2]	SK	TS	BJ
		Esters						
1	11.034	Isobutyl acetate	1028.6	110-19-0	32.58±10.55	54.70±34.16	n.d.	772.18±223.33
2	11.861	Butanoic acid, ethyl ester	1047.1	105-54-4	270.15±133.82	519.21±299.81	1287.09±290.11	678.94±194.95
3	13.246	Acetic acid, butyl ester	1078.1	123-86-4	101±46.20	198.45±65.11	3182.54±667.01	1532.70±386.43
4	15.102	1-Butanol,2-methyl-acetate	1118.6	624-41-9	1636.21±95.54	4869.11±781	10.26±1.47	6725.26±450.83
5	20.148	Hexanoic acid, ethyl ester	1225.5	123-66-0	56.53±51.22	43.30±27.12	252.95±29.39	678.44±73.97
6	22.094	Acetic acid, hexyl ester	1267.2	142-92-7	169.15±254.99	177.74±212.46	721.18±95.33	655.80±93.47
7	23.697	3-Hexenoic acid, ethyl ester	1301.6	2396-83-0	51.92±20.68	34.93±21.58	323.07±46.29	165.24±25.51
8	25.729	Propanoic acid, 2-hydroxy-, ethyl ester	1346.8	97-64-3	836.59±72.24	7197.52±917.67	488.35±79.47	56780.39±6998.80
9	29.466	Octanoic acid, ethyl ester	1432	106-32-1	297.74±222.44	90.81±52.27	552.86±163.68	734.75±97.37
10	30.456	Formic acid, heptyl ester	1455.5	112-23-2	109.26±17.14	12.54±2.02	30.02±4.36	72.41±1.90
11	32.078	Acetic acid, diethoxy-, ethyl ester	1494.1	6065-82-3	20.65±2.58	2.42±0.56	5.86±1.22	9.72±2.51
12	32.371	Pentanoic acid, 2-hydroxy-, ethyl ester	1501.1	6938-26-7	86.61±5.62	60.82±6.66	206.00±38.79	106.45±16.92
13	35.694	Propanedioic acid, diethyl	1581.5	105-53-3	14.99±2.35	14.56±3.42	74.47±13.46	61.24±7.71

		ester						
14	37.49	2-Furancarboxylic acid, ethyl ester	1626.8	614-99-3	146.23±19.47	77.45±13.24	173.04±19.50	118.48±3.79
15	37.497	Benzoic acid, methyl ester	1627	93-58-3	28.98±4.23	25.14±6.95	168.02±14.07	105.56±1.66
16	37.912	Butanoic acid, 4-hydroxy-	1637.7	591-81-1	19.67±0.42	61.07±8.77	6.76±1.68	13.11±3.15
17	37.960	Decanoic acid, ethyl ester	1639	110-38-3	26.79±18.04	4.91±2.61	3.48±0.30	23.26±2.89
18	39.211	Benzoic acid, ethyl ester	1671.4	93-89-0	10893.07±2276.44	2805.80±1173.90	7084.82±593.03	6946.64±210.61
19	39.533	Butanedioic acid, diethyl ester	1679.7	123-25-1	444.18±69.73	1877.89±435.22	2397.80±351.25	2998.34±647.42
20	41.580	Acetic acid, phenylmethyl ester	1734.4	140-11-4	283.90±26.95	10.20±2.34	13.07±1.35	56.16±3.19
21	43.379	Methyl salicylate	1783.3	119-36-8	40.73±8.93	60.23±24.11	83.01±8.18	57.44±7.47
22	43.671	Benzeneacetic acid, ethyl ester	1791.3	101-97-3	46.55±5.69	54.92±15.9	143.76±8.85	192.12±16.54
23	44.796	Acetic acid, 2-phenylethyl ester	1822.7	103-45-7	26.84±7.89	36.42±8.19	63.22±10.91	852.54±63.77
24	45.687	Dodecanoic acid, ethyl ester	1847.9	106-33-2	5880.55±491.68	945.87±159.01	1460.2±146.38	1231.66±251.82
25	47.069	β-Phenylethyl butyrate	1886.9	103-52-6	3.03±2.69	3.37±1.36	11.30±0.88	4.05±0.39
26	52.654	Tetradecanoic acid, ethyl ester	2050.7	124-06-1	1.75±0.28	n.d.	2.48±0.38	3.74±2.65
27	56.549	Benzoic acid, 4-ethoxy-, ethyl ester	2165.4	23676-09-7	26.26±16.43	16.65±3.67	116.07±35.85	126.18±36.16
28	59.06	Hexadecanoic acid, ethyl	2239.3	628-97-7	2.37±0.21	1.87±0.31	5.69±1.08	n.d.

		ester						
		Alcohols						
29	12.572	1-Propanol	1063	71-23-8	7408.91±2398.69	10142.67±530.72	39.89±5.89	3718.27±1495.50
30	14.447	1-Propanol, 2-methyl-	1104.7	78-83-1	10.80±10.67	15.41±34.36	6.43±0.04	n.d.
31	17.391	1-Butanol	1166.9	71-36-3	4301.15±257.78	5592.63±4470.85	2820.84±1625.61	3501.41±167.04
32	19.787	1-Butanol, 2-methyl-	1217.8	137-32-6	10322.75±883.28	16814.83±752.95	77.32±11.84	43810.51±3137.75
33	26.043	1-Hexanol	1353.8	111-27-3	2807.43±373.53	2772.54±230.25	5906.19±751.75	3648.79±172.19
34	30.238	1-Octen-3-ol	1450.4	3391-86-4	51.91±4.93	12.78±2.64	79.79±14.94	117.05±36.29
35	31.915	1-Hexanol, 2-ethyl-	1490.3	104-76-7	1.58±0.1	2.95±0.27	9.80±0.83	9.38±1.11
36	34.001	2,3-Butanediol	1540.6	513-85-9	2072.58±1118.24	39396.21±8480.69	77140.16±10619.77	77182.37±7268.29
37	34.690	1-Octanol	1557.2	111-87-5	53.00±7.62	17.26±3.86	51.87±3.92	128.55±14.30
38	37.037	2-Octen-1-ol, (E)-	1615.1	18409-17-1	4.68±1.11	2.65±0.73	13.52±0.94	16.73±1.61
39	38.733	1-Nonanol	1659	143-08-8	6.50±0.95	0.79±1.37	5.76±0.79	16.93±3.66
40	48.100	Phenylethyl Alcohol	1916.7	60-12-8	931.44±170.17	4776.34±901.97	59.38±15.05	22534.99±4601.87
41	48.680	Benzeneethanol, β-methyl-	1933.7	1123-85-9	n.d.	n.d.	n.d.	9.83±3.09
		Aldehydes and Ketones						
42	31.085	Furfural	1470.5	98-01-1	7.79±2.83	280.29±49.91	161.64±31.79	585.19±382.95
43	33.648	Benzaldehyde	1532	100-52-7	2018.98±121.88	661.35±156.22	64.94±9.82	105.08±50.18
44	36.671	Benzaldehyde diethylacetal	1605.6	774-48-1	5.57±3.01	565.01±253.82	240.69±10.25	760.50±199.4
45	38.365	Benzeneacetaldehyde	1649.5	122-78-1	5.63±6.47	385.25±84.61	759.78±100.84	1060.79±131.22
46	29.569	1-Propanone, 1-(4-aminophenyl)-	1434.5	70-69-9	5.97±1.76	3.07±0.92	9.86±3.97	7.28±0.61

47	38.656	Acetophenone	1657	98-86-2	n.d.	n.d.	n.d.	n.d.
48	44.104	1-Butanone, 1-phenyl-	1803.2	495-40-9	4.28±0.9	0.62±1.07	n.d.	6.53±0.57
		Terpenes						
49	42.052	Naphthalene	1747.3	91-20-3	14.26±2.68	13.43±5.09	35.05±1.84	58.84±10.45
50	34.317	1,6-Octadien-3-ol,3,7 dimethyl-	1548.2	78-70-6	96.59±13.34	91.90±16.69	278.55±26.58	239.92±12.67
51	40.276	α-Terpineol	1699	98-55-5	54.31±10.31	44.86±13.56	76.58±4.74	57.61±8.61
		Others						
52	30.330	Acetic acid	1452.6	64-19-7	8704.87±309.62	4404.69±446.05	4479.98±613.05	11774.91±1768.72
53	35.205	Propanoic acid, 2-methyl-	1569.7	79-31-2	52.94±2.70	46.42±0.94	18.80±3.51	216.81±34.07
